# Sudden infant death syndrome and the diaphragm: Is there a link?

**DOI:** 10.1038/s41390-021-01640-1

**Published:** 2021-07-05

**Authors:** Pontus M. Siren

**Affiliations:** Independent Researcher, Crassier, Switzerland

An April 6 correspondence in *Pediatric Research* draws attention to the fact that from over 12,500 sudden infant death syndrome (SIDS) articles on PubMed, only 59 focus on the diaphragm.^1^ The existing research offers no compelling reasons to exclude the diaphragm from SIDS research. Rather, the question arises: Is there evidence to suggest that the diaphragm should be investigated? In this context, at least seven research areas warrant our attention:

1. Pathogenic gene variants of SCN4A that encode for NaV1.4, a skeletal muscle voltage-gated sodium channel that is crucial for the generation of action potentials and excitation of muscle, are overrepresented in infants who died of SIDS compared with ethnically matched controls. These functionally disruptive SCN4A variants can impair the ability of respiratory muscles to respond to hypoxia.^2^
2. Elevated levels of vascular endothelial growth factor in the cerebrospinal fluid of SIDS infants indicate that they experience one or more hypoxic events over several hours to days before death.^3^ Hypoxia exacerbates diaphragm and abdominal muscle fatigability and can impair the diaphragm’s ability to generate force.^4^
3. There is a strong correlation between SIDS and nonlethal infections. It is well known that infections can produce severe respiratory muscle weakness in adults, which is a major contributor to respiratory failure. Infections can reduce the diaphragm’s ability to generate force by as much as 50% in 24 h.^5^
4. The prone sleeping position is an important risk factor for SIDS. For an infant, the prone position significantly increases the work of breathing and decreases respiratory muscle endurance.^6^
5. Hyperthermia is independently associated with an increased risk of SIDS as the 1990 Lancet editorial *Prone, hot, and dead* explains.^7^ It is well known that hyperthermia increases the workload of the respiratory muscles.^8^
6. SIDS is associated with rapid eye movement (REM) sleep as the original names of the syndrome, cot and crib-death, suggest. Intercostal muscles show both phasic and tonic inhibition during REM sleep that renders them largely or totally inactive. The chest wall muscles are critical for ventilation in the infant with a pliable chest wall, and loss of muscle tone increases the diaphragm’s workload.^9^
7. The Collaborative Home Infant Monitoring Evaluation study that entailed the analysis of over 700,000 h of home monitoring of infants 6 months after birth, concluded: “We determined that events previously described as ‘pathologic’ are actually quite common, even in healthy term infants.”^10^ The study monitored apnea, bradycardia, and hypoxemia, indicating that serious respiratory events are relatively common in young infants.

Considering that SIDS likely has a respiratory origin, that diaphragm failure is a well-known terminal event in adults, and the evidence discussed above, the possible link between diaphragm failure and SIDS should be investigated.

## Disclaimer

The work is original, it is not previously published, and is not submitted for publication or consideration elsewhere.
